# Does late gadolinium enhancement still have value for the RV? RV internal mechanical work, E_a_/E_max_ (VVC) and LGE as prognostic markers in pulmonary hypertension; a CMR study

**DOI:** 10.1186/1532-429X-18-S1-O136

**Published:** 2016-01-27

**Authors:** Amr E Abouelnour, Mark Doyle, Diane V Thompson, June A Yamrozik, Ronald B Williams, Moneal Shah, Siva K Soma, Srinivas Murali, Raymond Benza, Robert W Biederman

**Affiliations:** grid.413621.30000000404551168Cardiac MRI, Allegheny General Hospital, Pittsburgh, PA USA

## Background

### Objective

To investigate the impact of right ventricular (RV) internal work (IW), E_a_/E_max_ (VVC), and RV-late gadolinium enhancement (LGE) on outcome in pulmonary hypertension (PH).

LGE is known to be present within the RV insertion point (IP) and interventricular septum (IVS) in PH, but its prognostic role in such patients remains complex and potentially overestimated via 2D qualitative relative to 3D quantitative measures now available. However, RV-E_a_/E_max_, a measure of ventriculo-arterial coupling (VVC) and RV Internal Work (IW) when added to external cardiac work i.e. the P-V loop area denoting the RV's energetic demands, might fundamentally improve measures of PH prognosis as these metrics interrogate complex pulmonary physiology beyond the RV.

## Methods

CMR exams of 124 advanced PH patients (>50% WHO I; age=60 ± 13; 85 F, NYHA II-IV) referred to a large tertiary PH center were examined for RV volumetric and functional indices along with visual and quantitiative 2D and 3D RV-LGE. RHC were performed within 1 ± 2 months of the CMR. E_a_/E_max_ (VVC) was derived as RV end-systolic volume (ESV)/RVSV. IW was estimated as RVESV × (RV end-systolic pressure-RV diastolic pressure). Patients were followed from date of CMR for up to 5 years for MACE (death, hospitalized RV failure, initiation of parenteral prostacyclin, sustained ventricular arrhythmia or referral for lung transplantation).

## Results

Over one-third (48/124; 39%) of the patients had MACE by 18 months (1.6 ± 0.3 years). However, neither RVIP nor IVS LGE using visual assessment, 2D or 3D quantitation predicted MACE. The strongest predictor of MACE was RV IW versus multiparametric RV-LGE, VVC, mPAP, RV mass and RVEF.

## Conclusions

Neither a single time point visual, 2D or 3D RV-LGE metric can predict outcome in PH patients when compared with conventional or contemporary metrics of disease progression. In this largest 3D LGE assessment to date, physiologic, not anatomic parameters best represented MACE in patients with PH.

